# Association of C5L2 genetic polymorphisms with coronary artery disease in a Han population in Xinjiang, China

**DOI:** 10.18632/oncotarget.14353

**Published:** 2016-12-29

**Authors:** Ying-Ying Zheng, Xiang Xie, Yi-Tong Ma, Zhen-Yan Fu, Xiang Ma, Yi-Ning Yang, Xiao-Mei Li, Shuo Pan, Dilare Adi, Bang-Dang Chen, Fen Liu

**Affiliations:** ^1^ Department of Cardiology, First Affiliated Hospital of Xinjiang Medical University, Urumqi, 830054 P.R., China; ^2^ Coronary Heart Disease Laboratory, Xinjiang Key Laboratory of Cardiovascular Disease Research, Urumqi, 830054, P.R., China

**Keywords:** C5L2 gene, single nucleotide polymorphism, acylation-stimulating protein, coronary artery disease

## Abstract

**Background:**

C5aR-like receptor 2 (C5L2) has been identified as a receptor for the inflammatory factor Complement 5a (C5a) and acylation-stimulating protein (ASP). ASP binding to C5L2 leading to a net accumulation of TG stores and glucose transporter. The aim of the present study is to evaluate the association of the SNPs of C5L2 gene with coronary artery disease (CAD) in a Chinese population.

**Methods:**

We examined the role of the tagging single nucleotide polymorphisms (SNPs) of C5L2 gene for CAD using a case-control design. We determined the prevalence of C5L2 genotypes in 505 CAD patients and 469 age and sex-matched healthy control subjects of Han population.

**Results:**

There was significant difference in genotype distributions of rs2972607 and rs8112962 between CAD patients and control subjects. The rs2972607 was found to be associated with CAD in a dominant model (AA vs. AG + GG, *P*<0.001). Similarly, the rs8112962 was found to be associated with CAD in a dominant model (TT vs CT + CC, *P*=0.016). The difference remained statistically significant after multivariate adjustment (OR =1.401, 95% confidence interval [CI]:1.026~1.914, P=0.034; OR = 1.541, 95%CI:1.093~ 2.172, P=0.014; respectively).

**Conclusion:**

The results of this study indicate that both rs2972607 and rs8112962 of C5L2 gene are associated with CAD in a Han population of China.

## INTRODUCTION

Coronary artery disease (CAD) is a chronic complexity inflammatory disease, which is an interaction between environmental factors and genetics background. CAD becomes the number-one killer and account for one-third of all deaths in the world [1]. Ample evidences shown that inflammation throughout every stages of the atherosclerosis [1, 2]. Studies also showed that the estimation of Non-HDL cholesterol (Non-HDL), such as increased levels of triglyceride (TG), apolipoprotein B, and elevated fasting blood glucose (FBS) are important for the process of atherosclerosis, as well as CAD [3–5].

Anaphylatoxin and their receptors play key roles in the process of inflammatory. C3a and C5a are known as anaphylatoxin, which affect on the immune system [6] and is an important constituent of the cardiovascular disease(CVD). Acylation-stimulating protein (ASP), also named as C3a des-Arg, with increased level in obesity, insulin resistance, diabetes, CVD, and huyperthyroidism [7–10]. Also, ASP stimulates the glucose transport [11, 12] and synthesis of TG [13–16]. C5aR-like receptor 2 (C5L2) is one of the ASP receptors, which is involving a cascade of events including phosphorylation and receptor internalization [17]. C5L2 activation launched a downstream signaling transduction pathways that including protein kinase C activation and translocation, as well as glucose transporter [7, 18–19]. C5a (a powerful inflammatory factor) is a pro-inflammatory anaphylatoxin that interacts with two receptors: C5L2 and C5aR [20–24]. C5L2 is a potential ligand for both ASP and C5a, and has been implicated in many inflammatory diseases. A recent publication reported the mRNA expression of C5L2 in the aorta of apolipoprotein E deficient (Apoe−/−) mice. Varying levels of C5L2 depending on the stage of the disease were observed [25], which indicated that a similar trend could be seen in atherosclerosis. Therefore, the decreased activity of C5L2 may result in increasing susceptibility to CAD in individuals.

A novel mutation (S323I) of C5L2 gene, which was first reported by Marcil et al. [26] was found to be associated with familial combined hyperlipidemia in a French–Canadian family. Previously, we identified two new SNPs (698C > T and 901G > A), locating on codon 233 and codon 300, respectively, which are associated with CAD in both Chinese Han and Uygur population [27, 28]. However, the association of tag SNPs of C5L2 gene with risk of CAD remains unclear. In the present study, we aim to investigate the relation between tag SNPs and CAD in a Chinese Han population.

## RESULTS

### Baseline characteristics comparison between the two groups

The baseline characteristics of 974 participants including 505 CAD patients and 469 healthy controls were shown in Table 1. There are significant differences between the two groups in diabetes, drinking, glucose, Apo B and LP(a) between the two groups (all P<0.05). However, the distribution of age, sex, hypertension, smoking, body mass index (BMI) and serum concentration of TG, HDL and LDL between the two groups showed no significant differences (all P>0.05).

**Table 1:** Characteristics of the participants

Characteristics	Control (n=469)	CAD (n=505)	χ^2^ or *t*	*P* value
Age, mean (SD)	57.31 (10.27)	61.91 (9.94)	1.631	0.202
Sex, female (%)	159 (33.90)	169 (33.46)	0.019	0.470
Hypertension, n (%)	208(44.35)	239 (47.33)	0.868	0.368
Diabetes, n (%)	39 (8.31)	105(20.79)	30.045	<0.001
Smoking, n (%)	47 (10.02)	47(9.30)	0.142	0.745
Drinking, n (%)	42 (8.96)	27 (5.34)	4.811	0.033
BMI, mean (SD)	25.90(3.40)	25.50 (3.277)	0.896	0.344
Apo B, mean (SD)	0.84(0.23)	1.02(2.87)	4.033	0.045
Lipoprotein(a), mean (SD)	170.42(149.69)	203.53(190.51)	17.673	<0.001
TG, mean (SD)	1.94 (1.54)	2.87 (13.71)	3.460	0.06
TC, mean (SD)	4.35 (1.03)	4.27 (1.15)	2.907	0.089
HDL-C, mean (SD)	1.13 (0.35)	1.53(7.77)	3.716	0.054
LDL-C, mean (SD)	3.20 (13.64)	3.69 (13.09)	0.999	0.318
Glucose, mean (SD)	5.56 (1.77)	6.25 (2.60)	35.393	<0.001

### C5L2 genotypes distribution between the two groups

Table 2 showed the distribution of genotypes and alleles of tagSNPs of C5L2. The genotypes distributions for both CAD patients and controls were in line with the predicted Hardy-Weinberg equilibrium (H-WE) values (data not shown). There are significant differences between the CAD and the control group in the distribution of rs2972607 genotypes *(P*= 0.047), as well as a dominant model (AA vs GG + AG, *P*< 0.001), an additive model (AG vs AA + GG, *P*= 0.009) and an allelic model (*P*= 0.041), but no significant difference in a recessive model (GG vs AA + AG, *P*=0.440). Similarly, there are significant differences between the two groups in the distribution of rs8112962 genotypes (*P*= 0.014), as well as a dominant model (TT vs CC + CT, *P*= 0.016), an additive model (CT vs TT + CC, *P*= 0.005) and an allelic model (*P*< 0.001), but no significant difference in a recessive model (CC vs TT + CT, *P*=0.142).

**Table 2:** Genotype and Allele distributions in patients with CAD and control participants

variant	rs2972607			variant	rs8112962		
	CAD n(%)	control n(%)	P value		CAD n(%)	control n(%)	P value
genotype				genotype			
A/A	367(72.67)	369(78.68)	0.047	T/T	390(77.23)	389(82.94)	0.014
A/G	132(26.14)	92(20.26)		C/T	112(22.18)	73(15.57)	
G/G	6(1.19)	8(1.71)		C/C	3(0.59)	7(1.49)	
Dominant model				Dominant model			
AA	140(27.72)	369(78.68)	<0.001	TT	390(77.23)	389(82.94)	0.016
AG+GG	365(72.28)	100(21.32)		CT+CC	115(22.77)	80(18.06)	
Recessive model				Recessive model			
GG	7(1.19)	8(1.71)	0.440	CC	3(0.59)	7(1.49)	0.142
AG+AA	499(98.81)	461(98.29)		CT+TT	502(99.41)	462(98.51)	
Additive model				Additive model			
AG	132(26.14)	92(20.26)	0.009	CT	112(22.18)	73(15.35)	0.005
AA+GG	372(73.86)	377(80.74)		CC+TT	393(77.82)	397(84.65)	
Allele				Allele			
A	866(85.74)	830(88.49)	0.041	T	892(88.32)	851(90.72)	<0.001
G	144(14.26)	108(11.51)		C	118(11.68)	867(9.28)	

As shown in Table 3, for rs2972607, after adjustment of confounding factors such as plasma concentration of lipoprotein(a), diabetes, hypertension, drinking, and smoking habit using multivariable logistic regression analysis, the association remained in a dominant model (*OR* =1.401, 95%*CI*:1.026~1.914, *P*=0.034). Similarly, for rs8112962, after multivariable adjustment, the difference remained significant in a dominant model (*OR* = 1.541, 95%*CI*:1.093~ 2.172, *P*=0.014).

**Table 3:** Multiple logistic regression analysis for CAD patients and control subjects

	Factors	*B*	*S.E*.	*Wald*	*P*	*OR*	*95% CI*
rs2972607	Dominant model	0.337	0.159	4.499	0.034	1.401	1.026-1.914
	Diabetes	1.044	0.21	24.724	<0.001	2.84	1.882-4.285
	smoking	0.591	0.333	3.156	0.076	1.806	0.941-3.466
	drinking	−1.049	0.39	7.237	0.007	0.35	0.163-0.752
	lipoprotein(a)	0.001	0.000	6.369	0.012	1.001	1-1.002
	Constant	−1.275	0.417	9.352	0.002	0.279	
rs8112962	Dominant model	0.432	0.175	6.077	0.014	1.541	1.093-2.172
	Diabetes	1.03	0.211	23.891	<0.001	2.801	1.853-4.233
	smoking	0.579	0.333	3.023	0.082	1.783	0.929-3.424
	drinking	−1.025	0.39	6.917	0.009	0.359	0.167-0.77
	lipoprotein(a)	0.001	0.000	6.154	0.013	1.001	1-1.002
	Constant	−4.686	1.735	7.299	0.007	0.009	

## DISCUSSION

In the present study, we found C5L2 genetic polymorphisms were associated with CAD in a Chinese population. This is the first report to clarify the relation between tagging SNPs of C5L2 gene and CAD risk.

CAD is a chronic inflammatory Disease. A previous study has shown that TG/HDL-C ratio is a significant predictor of cardiovascular disease [29]. Lipids and inflammation are considered to play an important role in the pathogenesis of atheromatous plaque [3]. Lipoprotein(a) [Lp(a)] also correlates with CAD with insulin resistance and T2DM [30]. Both CAD and T2DM are multifactorial diseases in which hereditary and environmental factors both contribute to their etiology. Therefore, the association of genetic polymorphisms with CAD was paid attention to recently.

C5L2 is a potential ligand for both ASP and C5a. The level of circulated ASP is higher in CAD, hypertriglyceridemia [31], as well as obese subjects than normal controls [32], and plays a metabolic mediation role in atherosclerosis and T2DM. ASP through its receptor C5L2 can enhances lipid storage in adipocytes through stimulation of glucose uptake, fatty acid esterification and lipoprotein lipase activity [14]. In animal models(mice), ASP-C5L2 pathway can increased food intake may contribute to altered energy metabolism [33].

C5L2 is also a receptor of complement (C)5a. Evidences show that inflammation is a key process in the pathogenesis of atherosclerosis and is associated with acute cardiovascular events. Recent evidence suggested that C5a involves in adipocytes metabolism, which based on the effects on lipogenesis, glucose uptake, and lipolysis banding its C5L2 receptor [34]. C5a is an independent risk factor for nonspecific inflammation, which indicates that plasma C5a may be a prognostic prediction rather than markers of acute phase response in advanced atherosclerosis.

We genotyped the tag SNPs (rs2972607 and rs8112962) and investigate the relation between C5L2 genetic polymorphisms and CAD. We found significant difference between the CAD and the control group in genotype distributions of rs2972607 and rs8112962. After multivariate adjustment of confounding factors the significant difference was retained. A candidate gene approach is usually used to investigate the relation between genetics and CAD. The C5L2 gene is located on chromosome 19q13, which identified to be associated with familial combined hyperlipidemia, T2D and CAD by genome-wide scan studies) [35, 36]. Furthermore, previous studies showed that C5L2 plays an important role in insulin resistance, lipid metabolism and sepsis in a C5L2 KO mice model [37–40].

Previously, we reported two novel SNPs were associated with T2DM and CAD and were not modified by the concentration of HDL-C [27, 28]. Michel et al. [26] also identified a novel variant (S323I) in the C5L2 gene which was associated with familial combined hyperlipidemia in a French–Canadian family. These previous studies were in line with the present studies.

In conclusion, the present data suggest that common genetic polymorphisms of C5L2 gene are associated with CAD. However, our results need to be verified by future large sample size, multicenter case-control study.

## MATERIALS AND METHODS

### Ethical approval of the study protocol

All the participants are informed consent of this study protocol and sign an informed consent. An approval of the Ethics Committee of the First Affiliated Hospital of Xinjiang Medical University (Xinjiang, China) was obtained. It was conducted in accordance with the guidelines of the Helsinki Declaration.

### Subjects

Five hundred and five Han patients diagnosed with CAD at the First Affiliated Hospital of Xinjiang Medical University from January 2009 to December 2012 were recruited. Diagnosed with CAD based on the coronary angiography, the presence of at least one significant coronary artery stenosis of > 50% luminal diameter. Patients were excluded if they had congenital hypercoagulable status with proven disease-limiting life expectancy or had abused cocaine. Data and information about traditional coronary risk factors (include hypertension, diabetes as well as smoking) and other biochemical indicators were collected from all participants and a questionnaire of their family history of some chronic diseases.

The control subjects were comprised of 469 subjects selected from the Cardiovascular Risk Survey (CRS) [27, 28]. This consists of 14,618 subjects and is a multiple-ethnic, community-based, cross-sectional study designed to investigate the prevalence, incidence, and risk factors for CVD and to determine the genetic and environmental contributions to atherosclerosis, CAD and cerebral infarction (CI) of in the Han, Uygur, and Kazakh population in Xinjiang (west China) between June 2007 and March 2010. Individuals were excluded in who had underwent heart bypass surgery, coronary stenting, had history of myocardial infarction, CAD, electrocardiographic signs of CAD, and relevant valvular abnormalities in echocardiograms.

### Biological and lifestyle measurements

We collect five milliliters (5 ml) of peripheral venous blood from all the participants in an ethylene diamine-tetra acetic acid (EDTA) vial after at least 8 hours of fasting. Genomic DNA was extracted from the peripheral blood leukocytes by phenol and chloroform extraction using a whole blood genome extraction kit (Beijing Bioteke Corporation, Beijing, China. (http://bioteke.technew.cn) as previously described [41, 42]. We measured the serum concentration of uric acid, total cholesterol, TG, blood urea nitrogen (BUN), creatinine (Cr), low density lipoprotein (LDL), high density lipoprotein (HDL), Lipoprotein(a) [LP (a)], apolipoprotein B (Apo B) and fasting glucose using chemical analysis equipment (Dimension AR/AVL Clinical Chemistry System, Newark, NJ) in the Clinical Laboratory Department of the First Affiliated Hospital of Xinjiang Medical University as described previously [27, 41, 42].

### Genotyping of C5L2 gene

**Figure 1:**
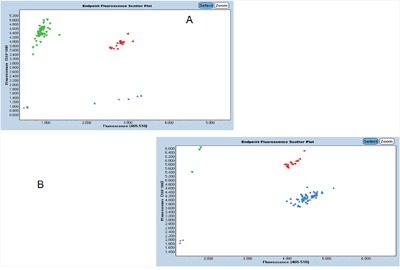
Determination of genotype **A**. For rs2972607, the AA genotype shows as green, the AG genotype shows as red, the GG genotype shows as blue. **B**. For rs8112962, the CC genotype shows as green, the CT genotype shows as red, the TT genotype shows as blue.

The ABI 7900 real-time PCR system (Applied Biosystems) for genotyping C5L2 gene. During the experiment, there are 4 blank control in each 96 sets. Using Haploview 4.2 software and international HapMap Project website phase I &II data base (http://www.hapmap.org), we obtained two tag SNPs: SNP1 (rs2972607) and SNP2 (rs8112962) by using minor allele frequency (MAF) ≥0.05 and linkage disequilibrium patterns with r^2^≥0.8 as a cutoff. Amplification was performed in 6ul volume, including 1ul of DNA, 3ul of Master Mix, 0.05ul of Probe(40X), MiliQ add to 6ul. Real time PCR was performed according to the protocol as follows: After an initial holding step of Pre-heatat 95°C for 10min. Samples were cycled 20 times at 95°C for 10s, at 65°C -55°C for 15s (Each loop drops by 0.5 °C), at 60°C for 15s. Then the samples were cycled 25 times at 95°C for 10s, at 60°C for 30s. pre/post read (Starting from 60°C at 0.06 °C /s was slowly warmed to 61 °C, and collecting the fluorescence value.). Cooling (cool at 40 °C to maintain). The results determination of genotype were shown in Figure 1.

### Statistical analyses

All analyses were performed by using SPSS version 17.0 (SPSS, Chicago, IL, USA). Hardy-Weinberg equilibrium result is reported by Chi-square analysis. Measurement data are shown as means ±SD, and the differences between CAD patients and control subjects were evaluated by independent-sample t-test. Differences in enumeration data between CAD patients and control subjects were analyzed using the chi-square test, as were differences in distributions of genotypes and alleles between CAD patients and control subjects. Logistic regression analyses were used to assess the contribution of the major risk factors. A P value less than 0.05 was considered significant.
